# Contrasting Winter Versus Summer Microbial Communities and Metabolic Functions in a Permafrost Thaw Lake

**DOI:** 10.3389/fmicb.2019.01656

**Published:** 2019-07-16

**Authors:** Adrien Vigneron, Connie Lovejoy, Perrine Cruaud, Dimitri Kalenitchenko, Alexander Culley, Warwick F. Vincent

**Affiliations:** ^1^Département de Biologie, Université Laval, Quebec, QC, Canada; ^2^Centre d’Études Nordiques, Takuvik Joint International Laboratory, Université Laval, Quebec, QC, Canada; ^3^Institut de Biologie Intégrative et des Systèmes, Université Laval, Quebec, QC, Canada; ^4^Québec Océan, Université Laval, Quebec, QC, Canada; ^5^Département de Biochimie, de Microbiologie et de Bio-Informatique, Université Laval, Quebec, QC, Canada

**Keywords:** MAGs, microbial diversity, metagenomes, methane, permafrost, thermokarst, winter limnology

## Abstract

Permafrost thawing results in the formation of thermokarst lakes, which are biogeochemical hotspots in northern landscapes and strong emitters of greenhouse gasses to the atmosphere. Most studies of thermokarst lakes have been in summer, despite the predominance of winter and ice-cover over much of the year, and the microbial ecology of these waters under ice remains poorly understood. Here we first compared the summer versus winter microbiomes of a subarctic thermokarst lake using DNA- and RNA-based 16S rRNA amplicon sequencing and qPCR. We then applied comparative metagenomics and used genomic bin reconstruction to compare the two seasons for changes in potential metabolic functions in the thermokarst lake microbiome. In summer, the microbial community was dominated by Actinobacteria and Betaproteobacteria, with phototrophic and aerobic pathways consistent with the utilization of labile and photodegraded substrates. The microbial community was strikingly different in winter, with dominance of methanogens, Planctomycetes, Chloroflexi and Deltaproteobacteria, along with various taxa of the Patescibacteria/Candidate Phyla Radiation (Parcubacteria, Microgenomates, Omnitrophica, Aminicenantes). The latter group was underestimated or absent in the amplicon survey, but accounted for about a third of the metagenomic reads. The winter lineages were associated with multiple reductive metabolic processes, fermentations and pathways for the mobilization and degradation of complex organic matter, along with a strong potential for syntrophy or cross-feeding. The results imply that the summer community represents a transient stage of the annual cycle, and that carbon dioxide and methane production continue through the prolonged season of ice cover via a taxonomically distinct winter community and diverse mechanisms of permafrost carbon transformation.

## Introduction

Northern landscapes are experiencing rapid change due to climate warming, with large scale thawing and erosion of permafrost soils in many regions (Oliva and Fritz, 2018). Around half of global soil carbon is estimated to be stored in permafrost (Schuur et al., 2015). This organic carbon is derived from the remnants of plants, animals and microbes that accumulated during warmer periods over the history of the Earth (Jansson and Taş, 2014; Schuur et al., 2015). Accelerated thawing is beginning to unlock these reserves along with indigenous permafrost microorganisms that were preserved in a dormant state (Graham et al., 2011; Mackelprang et al., 2017), and this old organic matter is now progressively decomposed by microbial activities. Shotgun metagenomic analysis of the microbiome of thawed subarctic soils has indicated a broad enzymatic capability to break down complex polymers that are found in permafrost (Woodcroft et al., 2018). The net result is the release of greenhouse gasses to the atmosphere (N_2_O, CO_2_ and CH_4_), and a positive feedback to climate warming (Schuur et al., 2015; Matveev et al., 2016). Decomposition and greenhouse gas emission rates from northern soils are variable (Parmentier et al., 2017), and some of the highest CH_4_ emissions of the permafrost region have been measured in thermokarst lakes and ponds (Matveev et al., 2016). These open waters form by thawing of ice-rich permafrost, which causes surface collapse and the creation of basins that fill with meltwater and precipitation. These lakes represent a quantitatively important type of aquatic ecosystem in Arctic and subarctic regions (Bouchard et al., 2014; Farquharson et al., 2016) and are viewed as biogeochemical hotspots in the permafrost landscape (Deshpande et al., 2016). Calculations suggest that the rapid thaw of sediments beneath thermokarst lakes by the latent heat of the water body could more than double the rate of carbon release from these abundant northern lakes by the end of the century (Walter Anthony et al., 2018).

Microbial communities in thermokarst lakes are reported to be similar to communities in other freshwaters, but enriched in methane cycling microorganisms (Crevecoeur et al., 2015, 2016; Comte et al., 2016) and uncultured bacterial taxa that are poorly resolved with standard primer sets (Wurzbacher et al., 2017). Thermokarst lake studies have been mostly carried out in summer, and microbial community composition during this open water period is strongly influenced by atmospheric exchange, meteorological conditions and exposure to sunlight, as in other freshwater ecosystems (Bertilsson et al., 2013). In contrast, all of these factors are absent or strongly reduced as soon as freeze-up occurs each winter. During this period that covers 8 or more months of the year in the North, ice and snow cover acts as an insulating barrier to atmospheric exchange and sunlight, compounded by short day lengths in winter. The ice cover leads to a rapid depletion of oxygen under the ice (Deshpande et al., 2017), while maintaining temperatures above freezing in the water column below the ice.

Methane production in winter is common in ice-covered lakes in temperate regions (Ricão Canelhas et al., 2016; Denfeld et al., 2018), and methane bubbles can become trapped in the ice of northern lakes (Langer et al., 2015). Even in temperate systems, the microbial community composition and metabolism under ice have been largely neglected compared to ice-free periods, despite evidence that the microbial community structure can differ substantially from summer to winter (Bertilsson et al., 2013). However, temperate lakes may not be an accurate guide to the functioning of thermokarst ecosystems. The summer primary production that occurs during a much shorter season of ice-free conditions in Arctic regions may not be sufficient to support the overwintering microbial communities, potentially leading to minimal biological activities and a rundown of microbial biomass by the end of the winter. Conversely, the abundance of terrestrially derived permafrost organic carbon (Wauthy et al., 2018), coupled with the prolonged anoxic conditions during winter (Deshpande et al., 2017), could be sufficient for an alternate community to develop during this time of year.

The aim of the present study was to compare the winter versus summer microbial communities of a thermokarst lake, and to evaluate two competing hypotheses: dormancy or downshift of the microbial community in winter toward lower population size and activity, versus a functional reassembly of the community without net loss of abundance or activity. We sampled a subarctic thermokarst lake, firstly during open water conditions in late summer, and then in late winter after several months of ice cover. To address the question of how microbiome structure and function differ between the two seasons, we characterized the microbial communities in summer and winter by DNA- and RNA-based 16S rRNA amplicons and quantitative PCR (qPCR), followed by construction of shotgun metagenomes. The analysis included evaluation of the metabolic pathways potentially involved in the transformation of permafrost organic carbon, and their seasonal persistence or change.

## Materials and Methods

### Site Description

The Sasapimakwananisikw (SAS) River Valley is located in the sporadic permafrost zone of subarctic Quebec, around 10 km southwest of the village Whapmagoostui-Kuujjuarapik, Quebec, Canada. The region experiences contrasting seasons, with a winter minimum air temperature of −42°C and a summer maximum of 33°C (CEN, 2017). The SAS valley contains numerous palsas, which are raised permafrost mounds in the peatland, and associated thermokarst lakes and ponds of different ages that formed over the last 60 years due to rapid permafrost thawing (Payette et al., 2004; Vincent et al., 2017). Lake SAS2A (55°13.160′ N, 77°41.806′W) is a circular thermokarst lake surrounded by semi-aquatic plants (notably *Carex aquatilis* and *Sphagnum* mosses) with an area of 196 m^2^ and a maximum depth of 2.8 m. Ice and snow conditions were monitored using automated cameras and sensors installed on the edge of the lake. In 2015–2016, the lake surface froze in mid-October 2015 and ice persisted until the end of May 2016 (details in Matveev, 2018 and CEN, 2017). The maximal snow (0.5 m) and ice (0.6 m) depth coincided with the time of our winter sampling in mid-March. Environmental data for lake SAS2A, including details on geochemistry (methane, carbon dioxide, dissolved organic carbon, sulfate and hydrogen sulfide concentrations) and limnological profiles (oxygen, pH, temperature, conductivity) measured at our two sampling times, are given in Matveev (2018) and Matveev et al. (2019), and are briefly described below.

**FIGURE 1:**
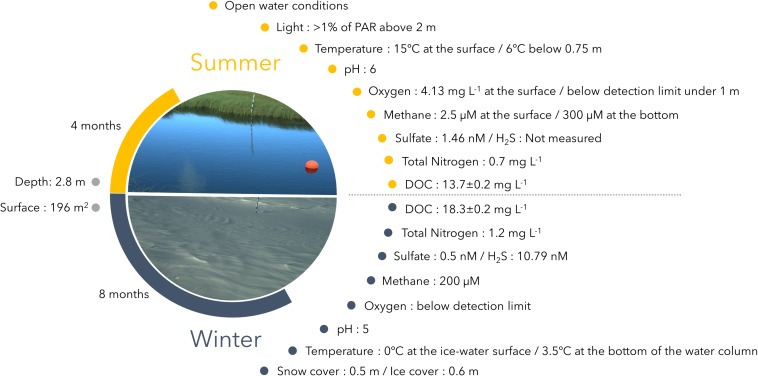
Study site characteristics with photographs taken at the two sampling times. Geochemical data (methane, nitrogen and DOC) are from Matveev et al. (2016, 2019). Sulfate data were provided by courtesy of J. Canario (University of Lisbon).

In summer 2015, lake SAS2A was darkly colored, with high concentrations of dissolved organic carbon (13.7 mg DOC L^–1^). This colored dissolved organic matter (CDOM) absorbs solar radiation, with <1% of the solar irradiance at below 0.7 m (Matveev et al., 2016) (Figure 1). The lake was slightly acidic (pH 6) in summer, with a drop in temperature, from 15°C at the surface to 6°C below 0.75 m. Although the surface waters are episodically mixed by wind, the lake is usually stratified throughout summer, with oxygen concentrations around 4.13 mg L^–1^ at the surface and values below the detection limit from 1 m to the water column bottom (Matveev, 2018). The lake is known to have high emission rates of CH_4_ (Matveev et al., 2016). Methane concentrations increased with depth, ranging from 2.5 μM at the surface to 300 μM at the bottom of the water column with 20 μM of methane at 0.5 m (Matveev et al., 2019). The CH_4_ emitted during summer has been ^14^C-dated at 1360 years before present, indicating breakdown of old permafrost-derived soil organic carbon (Matveev et al., 2016). Sulfate concentrations were low at the surface at around 1.46 nM, and below detection throughout the rest of the water column (Figure 1).

During winter 2015–2016, lake SAS2A was covered with ice and snow. At the time of sampling, water temperatures increased slightly with depth, from 0°C immediately beneath the ice to 3.5°C at the bottom of the lake. The lake was more acidic in winter (pH 5), and oxygen was below detection and methane concentrations high (around 200 μM) throughout the water column (Figure 1). Under-ice sulfate and hydrogen sulfide concentrations were up to 0.50 and 10.79 nM respectively (J. Canario, University of Lisbon, personal communication). DOC and total nitrogen concentrations were higher in winter than summer, up to 18.3 and 1.2 mg L^–1^, respectively (Figure 1; Matveev, 2018). Comparison of the upper water columns of three lakes (SAS2A and nearby SAS2B and SAS2C) indicated that SAS2A is likely to be representative of the humic-rich thermokarst waters throughout the valley, and that the chemical differences, including mean pH, methane and oxygen concentrations, are large and significant between seasons (all *t*-tests: *p* < 0.003; full profile data available in Matveev et al., 2019).

### Sample Collection and Nucleic Acid Extraction

Lake SAS2A was sampled in summer (24 August 2015) and winter (18 March 2016), at the same time as the limnological and geochemical characterization of the lake as shown in Figure 1 and described above. Sampling was with a 3–L Limnos Water sampler (KC, Denmark) that was previously washed with 10% HCl, rinsed with sterile MilliQ water, maintained closed until sampling and then rinsed three times with the lake water prior to sample collection. For the summer sampling, water was collected at 0.5 m below the surface (measured from the middle of the sampler), corresponding to the metalimnion/hypolimnion transition zone (oxycline), at three mid-lake sites. For the winter samples, snow cover (0.5 m) was removed and three independent 24 cm-diameter holes were drilled through the ice (0.6 m thickness) near the middle of the lake. The Limnos Water sampler was lowered into the ice holes and tripped immediately below the ice. For both the summer and winter campaigns, approximatively 300 mL of each triplicate water sample were filtered through separate 0.22 μm Sterivex filters and then stored below −50°C until nucleic acid extraction.

Nucleic acids (DNA and RNA) were extracted from the same Sterivex filters using Qiagen Allprep DNA/RNA Mini Kit with modifications as described in Cruaud et al. (2017). The Sterivex cartridges were opened and the membrane filters were cut into small pieces before the lysis steps. All steps of the nucleic acid extractions, from the opening of the filters to the nucleic acid resuspension in autoclaved, filtered and UV-treated water, were carried out in a sterile laminar flow cabinet. The DNA extracts were stored at −20°C until library preparation. For RNA extracts, two additional DNase steps (DNase I, Ambion, Foster City, CA, United States) were carried out to remove any trace of carried over DNA. The absence of DNA contamination was confirmed by amplification of 16S rRNA genes with bacterial primers using the RNA extracts (undiluted and diluted ten times) as template, with no product detected after 35 PCR cycles. The RNA was then immediately converted to cDNA using a High-Capacity cDNA Reverse Transcription kit (Applied Biosystems, Foster City, CA, United States) and stored as cDNA at −20°C until library preparation.

### Quantitative Polymerase Chain Reaction (qPCR) Analysis

The number of Bacteria and Archaea 16S rRNA genes in the samples from the two seasons were estimated using quantitative PCR (qPCR), with primers Bact1369f/Bact1492r and Arc787f/Arc1059r, respectively (Vigneron et al., 2013). Quantification was performed in triplicate with a range of template concentrations (0.5, 1, 1.5 ng of DNA) to compensate for any PCR inhibition. Amplification reactions were carried out in a 7500 Fast Real-Time system (Applied Biosystems) in a final volume of 25 μL using Brilliant III Ultra-Fast Master Mix (Agilent, Santa Clara, CA, United States), 0.5 μM of each primer and 5 μl of DNA template. qPCR conditions were as follows: 40 cycles of denaturation at 95°C for 15 s then annealing and extension at 60°C for 60 s. Standard curves were prepared in triplicate with dilutions ranging from 0.001 to 100 nM of DNA extracted from *Methylomonas methanica* (DSM25384) and *Methanosarcina acetivorans* (DSM2834), corresponding to 10^2^ to 10^6^ 16S rRNA gene copies per reaction. The R^2^ of standard curves obtained by qPCR were above 0.99, PCR efficiencies were above 90.5%, and melting curves showed no trace of non-specific amplifications. The qPCR results were expressed in terms of 16S rRNA gene numbers per milliliter of lake water.

### Illumina MiSeq Amplicon Library Preparation, Sequencing and Analysis

Microbial community composition of lake SAS2A in the two seasons was determined by high throughput sequencing of bacterial and archaeal 16S rRNA (cDNA) and 16S rRNA genes (DNA) using primers targeting the bacterial V4-V5 region (S-D-Bact-0516-a-S-18/S-D-Bact-0907-a-A-20; 460 bp product) (Klindworth et al., 2013) and the archaeal V1-V3 region (A27F/Arc518R; 500 bp), respectively (Teske and Sorensen, 2007). All PCR reactions were carried out following Vigneron et al. (2017). Amplicons were sequenced using an Illumina MiSeq v3 kit at the IBIS/Laval University, Plate-forme d’Analyses Génomiques (Québec, QC). Reads were assembled into single paired-end sequences and curated as detailed in a GitHub repository^1^. Taxonomic affiliations of the reads were determined with Mothur (Schloss et al., 2009) using BLAST against Silva database release 128 as reference (Pruesse et al., 2007).

### Metagenomic Library Preparation, Sequencing and Analysis

Three metagenomes were constructed for winter and summer samples (total of 6 metagenomes) using a Nextera XT Library Kit (Illumina, San Diego, CA, United States) according to the manufacturer’s recommendations at the CGEB - Integrated Microbiome Resource, Dalhousie University. Sequencing was performed using four lanes of an Illumina NextSeq system at the CGEB, Dalhousie University. Barcode and adapter sequences were removed from the metagenome data on-instrument using Illumina’s MiSeq Reporter software and the sequence data were exported as FASTQ files. Datasets were quality filtered using the Trimmomatic tool (Bolger et al., 2014), with default settings. Paired-end joining was done using FLASH2 (Magoč and Salzberg, 2011). The 16S rRNA genes (on average *n* = 30795 per sample) were isolated from metagenomic reads using REAGO 1.1 (Yuan et al., 2015), and taxonomic assignments were performed as for the 16S rRNA gene amplicons.

Each metagenome was assembled separately from paired-end reads after quality filtering using IDBA_UD (Peng et al., 2012). Assembled contigs and mapping files (BAM files) were uploaded to the Department of Energy Joint Genome Institute (DOE-JGI) IMG/MER analysis pipeline (Markowitz et al., 2009) for gene calling and functional annotation. To account for differences in sequencing depth between samples, metagenomes were normalized by multiple rarefaction (100 iterations) to the size of the smallest dataset (218503 genes). For metagenome assembled genome reconstruction, all quality filtered sequences were pooled and co-assembled using MEGAHIT (Li et al., 2015), which was computationally less expensive than other options and appropriate for our dataset. Read coverage of the contig was carried out using bwa-mem^2^, then contig binning was done using MetaBAT-1 and -2 (Kang et al., 2015) and Maxbin 2.0 (Wu et al., 2016) with contigs longer than 3000 bp. In addition, binning of the single sample-assembled contigs (longer than 3000 bp) was also performed using Vizbin (Laczny et al., 2015). The multiple binning tools increased the number of bins. Redundant bins obtained from the 4 binning algorithms were identified and removed using CheckM (Parks et al., 2015). The completeness and contamination level of the combined genomic bins were then evaluated using CheckM (Parks et al., 2015). Only bins with a contamination level under 5% and completeness above 50% were analyzed. Genetic composition of genomic bins was then explored using KEGG (Kanehisa et al., 2008) and MetaCyc (Caspi et al., 2014) based on genes identified by IMG/MER in the co-assembly. The results were manually checked and pathways were considered as present when 75% of the genes involved in the pathway were detected. The presence of specific pathways was also determined by detection of key genes (in brackets in Figure 4). The presence of genes for carbohydrate-active enzymes was investigated using dbCAN (Yin et al., 2012) and the CAZy database (Cantarel et al., 2009). Assembled metagenome data are available in IMG/MR under the following accession numbers: 3300022653, 3300022650 and 3300022591-4. Raw sequences were deposited in the NCBI public database under Bioproject PRJNA515027 and bin files are available in FigShare (doi: 10.6084/m9.figshare.8132546).

### Statistical Analyses

**FIGURE 2:**
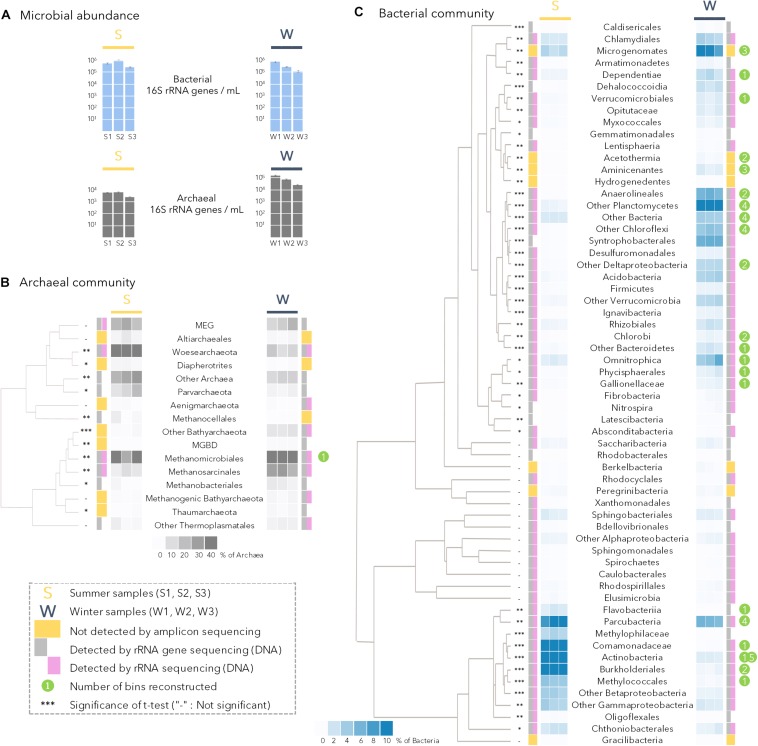
**(A)** Bacterial and archaeal abundance determined by quantitative PCR in all summer (S1, S2, S3) and winter (W1, W2, W3) samples. **(B)** Archaeal community composition in summer (S1, S2, S3) and winter (W1, W2, W3) samples. MBGD: Marine Benthic Group B; MEG: Miscellaneous Euryarchaeotal Group. **(C)** Bacterial community composition in summer (S1, S2, S3) and winter (W1, W2, W3) samples. Heat maps of the relative proportion of microbial lineages are based on 16S rRNA genes from metagenomic dataset. Clustering of the microbial lineage relative proportion (constructed by UPGMA with Euclidian distance on the correlation matrix generated using microbial lineage relative proportions) gather lineages with similar relative proportion in the samples. Gray rectangles (detected by PCR on DNA template) and purple rectangles (detected by PCR on cDNA template) indicates the detection of a given lineage by 16S rRNA (gene) amplicon sequencing whereas lineages with yellow squares were not detected by amplicon sequencing (details in Supplementary Figure 1). Differences in the relative proportion between summer and winter samples were evaluated by *t*-tests; the significance of the test is presented as: -, not-significant; ^*^p < 0.05; ^∗∗^*p* < 0.01; ^∗∗∗^*p* < 0.0005. The number of genomic bins reconstructed per lineage is indicated by the green circles.

Statistical analyses of the data set [Student *t*-test, NPMANOVA, similarity percentages breakdown procedure (SIMPER)], Bray-Curtis-based dissimilarity index calculations and correlation-based clustering were carried out according to recommendations of the Guide to Statistical Analysis in Microbial Ecology (Buttigieg and Ramette, 2014), using PAST software (Hammer et al., 2001). Correlation matrices were calculated using microbial lineage relative proportions, then clustering of microbial lineages shown in Figure 2 was determined by UPGMA based on Euclidian distances generated from these correlation matrices.

## Results

### Microbial Community Composition

The microbial community composition and abundance in lake SAS2A was determined by 16S rRNA (cDNA), rRNA gene (DNA) amplicon sequencing, quantitative PCR and metagenomic shotgun sequencing. Overall, there was no significant difference in the total concentration of 16S rRNA genes (Bacteria plus Archaea) between summer and winter (*t*-test, *p* = 0.85; Figure 2A). These values averaged around 4.8 ± 3 × 10^5^ 16S rRNA genes mL^–1^ for summer samples and for 4.3 ± 3 × 10^5^ 16S rRNA genes mL^–1^ for winter samples. However, the concentrations of archaeal gene copies were significantly greater (15 times higher; *t*-test, *p* < 0.001) in winter than in summer, with up to 6 × 10^4^ 16S rRNA genes mL^–1^ (Figure 2B). In summer, Archaea accounted for only 1% of the total prokaryotes in both the qPCR and metagenomic analyses, but 7% (qPCR) and 17% (metagenomes) in winter.

The winter archaeal community, determined from the 16S rRNA genes in the metagenomes, was mainly dominated by Methanomicrobiales (50% of the Archaea), Methanosarcinales (14%) and other co-varying potential methanogenic lineages and Bathyarchaeota (Figure 2B). In contrast, the archaeal community in summer had a lower contribution of Methanomicrobiales (22%), and a larger contribution of Woesarchaeota (34% of the Archaea), and Miscellaneous Euryarchaeal Group (MEG, 11%).

Analysis of metagenomic 16S rRNA genes showed that bacterial community composition also differed between winter and summer samples (Bray Curtis dissimilarity index: 73.27, NPMANOVA: *p* = 0.05). The summer bacterial community was dominated by Betaproteobacterial members of the Burkholderiales (27% of the bacterial 16S rRNA genes), *Comamonadaceae* (12%) and *Methylophilaceae* (3%); Actinobacteria (17%), Parcubacteria (10%) and other co-varying lineages of the Gammaproteobacteria including Methylococcales (Figure 2C). The relative proportions of these lineages were statistically greater in summer samples (*t*-tests, *p* < 0.005; bottom cluster in Figure 2C). In contrast, in winter samples, members of the Planctomycetes (15%), Chloroflexi (14% with *Anaerolineae*, *Dehalococcoidia* and Chloroflexi), Microgenomates (9%), Deltaproteobacteria as Syntrophobacterales (6.4%), and Omnitrophica (5%) had a significantly greater representation compared to summer samples (*t*-tests, *p* < 0.05; Figure 2C). Although the relative proportions of the Parcubacteria and Microgenomates were significantly different between seasons, the two phyla represented a major component of the bacterial community in all samples (Figure 2C). Finally, amplicon sequencing of the 16S rRNA gene using DNA and RNA templates showed that most of the detected lineages were potentially active, or at least maintained ribosomes in their cells in both summer and winter seasons (Figure 2C, **gray** and **pink** rectangles next to the heatmap).

### Overall Microbial Metabolic Potential

**FIGURE 3:**
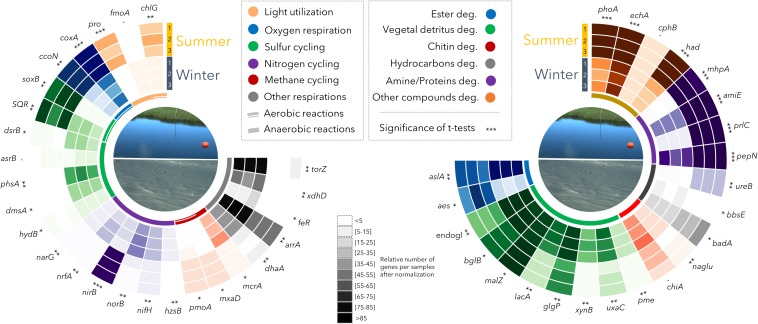
Relative proportion of metabolic genes identified in Summer (S1, S2, S3) and Winter (W1, W2, W3) samples. The relative abundance of selected genes was represented by a 10-interval shading, with darker shades indicating higher abundance. Significance of *t*-tests: -, not-significant; ^*^p < 0.05; ^∗∗^*p* < 0.01; ^∗∗∗^*p* < 0.0005. *chlG*: chlorophyll synthase; *fmoA*: bacteriochlorophyll A protein; *pro*: proteorhodopsin; *coxA*: cytochrome c oxidase subunit 1; *ccoN*: cytochrome c oxidase cbb3-type; *soxB*: sulfur-oxidizing protein; *sqr*: sulfide_quinone oxidoreductase; *dsrB*: sulfite reductase; *asrB*: anaerobic sulfite reductase; *phsA*: thiosulfate reductase/polysulfide reductase; *dmsA*: anaerobic dimethyl sulfoxide reductase; *hydB*: sulfhydrogenase; *narG*: nitrate reductase: *nrfA*: nitrite reductase (cytochrome c-552); *nirB*: nitrite reductase; *norB*: nitric oxide reductase; *nifH*: nitrogenase, *hzsB*: hydrazine hydrolase (Anammox); *pmoA*: methane monooxygenase; *mxaD*: methanol dehydrogenase; *mcrA*: methyl coenzyme M reductase; *dhaA*: haloalkane dehalogenase; *arrA*: arsenate respiratory reductase; *feR*: ferric-chelate reductase; *xdhD*: selenate reductase; *torZ*: trimethylamine-N-oxide reductase; *aslA*: arylsulfatase; *aes*: acetyl esterase; endogl.: endoglucanase; *bglB*: beta-glucosidase; *malZ*: alpha-glucosidase; *lacA*: beta-galactosidase; *glgP*: starch phosphorylase; *xynB*: xylanase‘; *uxaC*: glucuronate isomerase; *pme*: pectinesterase; *chiA*: chitinase; *naglu*: alpha-N-acetylglucosaminidase; *badA*: benzoate-CoA ligase; *bbsE*: benzylsuccinate CoA-transferase; *ureB*: urease; *pepN*: aminopeptidase; *prlC*: oligopeptidase; *amiE*: amidase; *mhpA*: (hydroxy-phenyl)propionate hydroxylase; *had*: haloacid dehalogenase; *cphB*: cyanophycinase; *echA*: enoyl-CoA hydratase; *phoA*: alkaline phosphatase. Soluble methane monooxygenase gene (mmoX) was only detected at very low levels in sample S1.

The shotgun metagenomes from summer (*n* = 3) and winter (*n* = 3) samples were used to explore the metabolic potential of the permafrost thaw lake. Up to 8.15 × 10^5^ genes with predicted functions were identified per sample (average: 7.16 × 10^5^ genes). These genes were distributed into at least 6500 different molecular-level functions (KEGG Orthology). The metagenomic datasets were normalized by multiple rarefaction (see Methods section) then compared. The metabolic potential for degradation of complex organic molecules (e.g., aromatic compounds, glycans, starch, cellulose, hydrocarbons) was present in all samples (Figure 3). In contrast, different potential energetic pathways were detected between winter and summer samples, notably in the methane and sulfur cycles (Figure 3). In the summer samples, which were collected from the oxycline, the potential for oxygen respiration and aerobic metabolism (sulfide, thiosulfate, methane and methanol oxidation) was predominant. The potential for light utilization through photosynthesis (*chlG*) and proton pumping with proteorhodopsin (*pro*) was also evident (Figure 3). In contrast, in the winter sampling, when no oxygen was detected in the water at the time of collection, a considerable potential for reductive metabolism was identified. The winter microbial community harbored the potential for sulfate (*dsrB*), sulfite (*asrB*), thiosulfate (*phsA*), dimethyl-sulfate (*dmsA*), polysulfide (*hydB*), nitrate (*narG*), nitrite (*nrfA*), selenate (*xdhD*), arsenate (*arrA*), trimethylamine oxide (*torZ*) and chelated iron (*feR*) reductions along with methanogenesis (*mcrA*) and dehalogenation (*dhaA*, Figure 3).

### Genomes Assembled From Community Metagenomes

**FIGURE 4:**
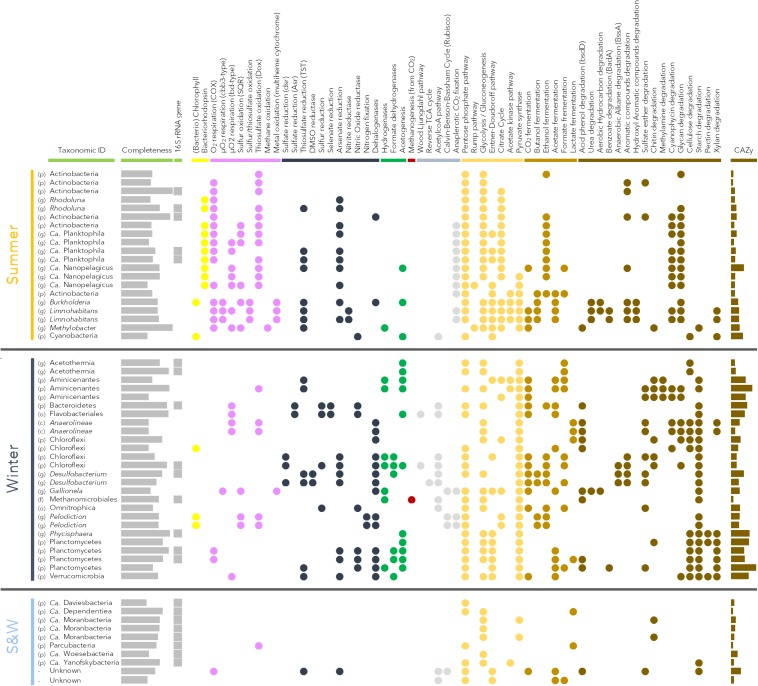
Metabolic pathways identified in reconstructed genomic bins with >50% completeness and <5% contamination level. Taxonomic identification of the bins was based on 16S rRNA sequences when present and on other phylogenetically informative marker genes when no 16S rRNA gene was present. First column indicates best taxonomic level assigned for the bins with (p): phylum; (o): order; (c): class; and (g): genus. Identification of the pathways was carried out with MetaCyc and the KEGG pathway mapping tool. Yellow dots indicate light utilization; purple: oxidative processes; blue: reductive processes; green: syntrophic metabolism; red: methanogenesis; gray: CO_2_ fixation; orange: carbon assimilation; light brown: fermentation; and dark brown: organic matter degradation. Results for specific pathways absent from the KEGG database (bacteriorhodopsin, metal oxidation, arsenate reduction) were identified by BLAST of the bins against in-house databases for these specific genes and manually checked. The number of carbohydrates-active enzyme genes (CAZy, max = 192 genes for Planctomycetes bin) was determined using dbCAN against the CAZy database.

Genomic bins corresponding to representative microbial lineages in the permafrost thaw lake were assembled, and metabolic capabilities were inferred from the genomic bins with >50% completeness and contamination levels of <5% (Figure 4). The recovered genomic bins exhibited contrasting metabolic potentials, which reflected their taxonomic affiliation and the seasonal environmental conditions of the thermokarst lake.

Genomic bins recovered from the summer samples were largely affiliated to taxa within the phylum Actinobacteria with 15 bins, including the genera Actinobacteria, *Rhodoluna* and *Ca.* Planktophila and *Ca.* Nanopelagicus (Figure 4). The potential for bacteriorhodopsin phototrophy and oxygen respiration was identified in most of the Actinobacteria genomic bins. Different types of cytochromes for oxygen respiration (types 1, cbb3 and bd) were detected. The potential for thiosulfate oxidation through thiosulfate:quinone oxidoreductase was also widespread in the Actinobacterial bins (Figure 4). Regarding carbon metabolism, the potential for degradation of cyanophycin and complex glycans was identified along with the pentose phosphate pathway, glycolysis and citrate cycle, which permit carbohydrate assimilation (Figure 4). In addition, the potential for anaplerotic CO_2_ fixation (phosphoenolpyruvate carboxylase and pyruvate carboxylase genes), ethanol fermentation, thiosulfate and arsenate reduction was also present in most of the Actinobacteria genomic bins. Three large genomic bins affiliated to Betaproteobacteria were also recovered (*Burkholderia* and *Limnohabitans*). Numerous oxidative metabolic functions were identified in these bins, including oxygen respiration, thiosulfate oxidation through the Sox pathway and metal oxidation via multiheme cytochrome C. A strong metabolic potential for degradation of various organic compounds, including urea, hydrocarbons and aromatic compounds was also identified (Figure 4). Most catabolic pathways (pentose phosphate pathway, glycolysis, Entner-Doudoroff pathway, acetate kinase pathway, citrate cycle) were also detected in these lineages. Genomic bins affiliated to *Methylobacter* (Gammaproteobacteria) and Cyanobacteria were also assembled. The capacity for thiosulfate reduction as well as methane oxidation was detected in the *Methylobacter* genomic bin, and photosynthetic functions were evident in the Cyanobacteria, as expected (Figure 4).

Genomic bins assembled from the winter samples were related to 13 different taxa, representing the dominant winter microbial lineages (Figures 2B,C, 4). The potential for hydrogen, acetate and pyruvate metabolism was widespread in the winter community. Planctomycetes genomic bins (*n* = 5) harbored a strong potential for organic matter degradation (cellulose, starch, xylan and pectin) associated with numerous genes for carbohydrate degradation (up to 192 carbohydrate-active enzymes (CAZy) genes per bin; Figure 4). The potential for ethanol fermentation, acetogenesis and organohalide and arsenate respiration was also detected in most Planctomycetes bins (Figure 4). Six genomic bins affiliated to Chloroflexi (Chloroflexi and *Anaerolineae*) were assembled. Different metabolic potentials were identified within this group. *Anaerolineae* bins showed the genetic potential for micro-oxygen and thiosulfate respiration, organohalide respiration and lactate fermentation coupled with the potential to degrade acid phenol, cyanophycin, glycans, cellulose and starch. Two other Chloroflexi bins had the potential for sulfate, arsenate and organohalide reduction, hydrogenotrophy, and various fermentations coupled to genes involved in hydrocarbon and aromatic compound degradations. In addition, another Chloroflexi bin showed the potential for anoxygenic phototrophy (Figure 4). Such potential for light utilization was also detected in two bins affiliated to Chlorobi (*Pelodiction*), where this potential was associated with sulfur and thiosulfate oxidation and CO_2_ fixation through the Calvin-Benson-Bassham cycle (Figure 4). The potential for thiosulfate reduction, hydrogenotrophy and acetogenesis was identified in two Aminicenantes genomic bins. A strong potential for complex carbon substrate degradation (130 CAZy genes per bin) was also present in Aminicentantes genomic bins, along with the potential for multiple fermentation pathways (Figure 4). Acetothelia related bins contained genes for acetogenesis and formate fermentation, but a limited potential for organic carbon degradation (Figure 4). Two genomic bins related to *Desulfobacterium* lineages showed the potential for sulfate, thiosulfate, DMSO, arsenate and organohalide reduction, numerous fermentations and degradation of hydrocarbons and aromatic compounds (Figure 4). The Bacteroidetes (Bacteroidetes and Flavobacteriales) bins harbored the potential for sulfate, sulfur and selenate reduction and micro-oxygen respiration, suggesting a facultative aerobic lifestyle. Finally, many genes for carbohydrate degrading enzymes were identified in all of these genomic bins (114 CAZy genes, Figure 4). Single bins of Omnitrophica, Verrucomicrobia, *Gallionella* were also assembled. Archaea were also represented with a genomic bin related to the Methanomicrobiales, with genes for hydrogenotrophic (H_2_:CO_2_) methanogenesis (Figure 4).

Finally, eight genomic bins were recovered from the Patescibacteria/candidate phyla radiation (*Parcubacteria*, *Ca.* Moranbacteria, *Ca.* Dependentiae and *Microgenomates*). These represented a substantial proportion of both winter and summer microbial communities, but had a limited number of genes with known functions. With the exception of a gene coding for a thiosulfate:quinone oxidoreductase in a *Parcubacteria* bin, no respiratory pathways were identified. The potential for simple carbohydrate assimilation through glycolysis or pentose phosphate pathway was detected, as well as the potential for lactate fermentation in *Parcubacteria* and *Ca.* Dependentiae. A few genes for carbohydrate degradation enzymes were also present in all Patescibacteria/CPR genomic bins (average of 30 CAZy genes; Figure 4).

## Discussion

In this study, we compared the winter and summer microbial communities of a thermokarst lake to evaluate whether the winter season induces dormancy of the microbial community or, in contrast, is the time of functional reassembly into a winter-active community. Contrary to the hypothesis of a microbial downshift or dormancy in winter, the qPCR comparison of the ice-covered and ice-free periods indicated no statistical change in total prokaryotic abundances between seasons, and in fact there was an order of magnitude increase in archaeal abundance (Figure 2A). Furthermore, the amplicon sequencing based on cDNA indicated that with few exceptions, all detected microbial lineages were potentially active or ready to respond to favorable environmental conditions (pink rectangles in Figures 2B,C, details in Supplementary Figure 1). For the two seasons, a large panoply of microorganisms was identified by the 16S rRNA gene and metagenomic sequencing, including Actinobacteria, Chloroflexi, Planctomycetes, Proteobacteria, candidate phyla, Methanomicrobiales and Woesarchaeota (Figures 2B,2C). Additional phyla, notably Microgenomates, Aminicenantes and Acetothermia, were solely identified by PCR-free metagenomic sequencing (yellow squares in Figure 2). These latter phyla collectively represented a third of the thermokarst lake microbial community in both summer and winter samples, yet were not detected in previous reports based on 16S rRNA gene amplicons (Crevecoeur et al., 2015; Wurzbacher et al., 2017). This may be due to the presence of introns in their 16S rRNA genes that precluded PCR amplification with conventional 16S rRNA gene primer sets (Brown et al., 2015). These results highlight the value of a metagenomic approach for thermokarst lake community composition analysis, and they show that SAS2A is a rich microbial habitat containing diverse taxa that likely maintain a substantial biomass and productivity throughout the year.

### Seasonal Contrasts

Consistent with the hypothesis of an alternate community developing under the ice, the clustering of the microbial lineages based on their relative abundance in the metagenomic dataset indicated a fundamental shift in community composition between summer and winter (NPMANOVA: *p =* 0.05; Figure 2). This major shift in taxonomic composition was accompanied by large differences in potential functioning of the community, with contrasting, and sometimes opposing, metabolic pathways between seasons (Figure 3).

The summer microbial community was dominated by bacteria (99% of the 16S rRNA genes from qPCR and metagenomes) with predominant members of the Actinobacteria and Betaproteobacteria lineages (Figure 2), as previously reported in other thermokarst lakes (Comte et al., 2016) and freshwater ecosystems in general (Newton et al., 2006, 2007). The recovery of multiple Actinobacteria genomic bins (Figure 4) would be consistent with micro-diversification in genomic composition, suggesting a strong diversity within this lineage. This diversity would facilitate rapid responses to environmental fluctuations (Neuenschwander et al., 2017) that would arise from near-surface stratification and periodic mixing in the thermokarst lake environment during summer. In contrast, the genomic bins of Betaproteobacteria suggested a different ecological strategy to that observed in Actinobacteria, with multiple pathways of energy acquisition, suggesting a metabolic versatility to cope with environmental fluctuations. Consistent with ample light and oxygen in the upper water column during summer (Figure 1), the overall metabolic capacities of the microbial community were centered on phototrophy, and aerobic metabolism (oxygen respiration and oxidation of methane, methanol, thiosulfate and H_2_S) coupled to the degradation of particulate and photodegraded organic matter (*mhpA* in Figure 3). Methane, methanol, H_2_S and thiosulfate oxidations are likely fuelled by the release of methane and other metabolic products from the sediments and the anoxic layer of the water column. This potential for methane oxidation and the larger CO_2_ emissions reported in summer supports the notion that the greenhouse gas balance of lake SAS2A is oriented toward net CO_2_ production during open water conditions (Laurion et al., 2010), and that the microbial community in the upper water column acts as a biofilter that dampens summer methane emissions, as previously suggested (Crevecoeur et al., 2017).

In the winter season, ice cover forms a diffusive barrier with the atmosphere, leading to the rapid (∼10 days after ice-cover formation) depletion of oxygen and anoxia in the water column (Figure 1; Deshpande et al., 2017). Consequently, reduced compounds produced in the anoxic bottom of the lake are not recycled by the oxidative processes observed during summer. The microbial community composition under the ice shifted to members of the Planctomycetes, Chloroflexi, Deltaproteobacteria, Candidate phyla and Methanomicrobiales (Figures 2A,B). The metabolic capacities of the winter community also reflected this seasonal shift, with various anaerobic pathways such as methanogenesis, consistent with higher methane concentrations in the water and the reduction of potential oxidants (Figure 3). Genomic bin reconstruction indicated that nitrate, sulfate, iron, arsenate, and selenate can be reduced by the winter community (Figure 4), potentially leading to a strongly reduced environment that would further allow the methanogens to flourish (Figure 2). These reduction pathways appeared to be coupled to complex organic matter degradation, fermentations, and the production and consumption of hydrogen, formate and acetate (Figures 3, 4). This suggests that syntrophy, defined as a metabolic interaction between dependent partners, and complementary metabolism (facultative cross-feeding) between methanogens and their putative syntrophic and fermentative partners (*Syntrophobacteraceae*, Chloroflexi*;* de Bok et al., 2004; Liang et al., 2015) are likely to be major ecological strategies of the winter microbial community. The net effect of such syntrophy and cross-feeding would be an enhanced capacity to degrade recalcitrant organic matter in the isolated and reduced environmental conditions of the ice-covered thermokarst lake. These results, coupled with the winter chemical conditions indicate a seasonal partitioning of the biogeochemical cycles in the thermokarst lake, where methane and potentially reduced molecules accumulate under ice, leading to a massive release of greenhouse gasses when the ice breaks up in spring (Deshpande et al., 2017). However, aerobic methanotrophic lineages, such as members of the Methylococcaceae were detected in winter (Figure 2C), suggesting that they might survive winter using alternate metabolic pathways such as thiosulfate or arsenate reduction and fermentations, as identified in their genomic bins (*Methylobacter* bin in Figure 4). This overwintering population of methanotrophs could potentially moderate atmospheric methane emissions from the lake in spring.

These results indicate that the thermokarst lake community has a high potential for rapid adjustments in response to episodic and seasonal changes in environmental conditions such as oxygen, temperature and mixing. An important consequence of such flexibility in composition and function is that analyses in summer of community structure, metagenomic features and microbial activities such as gas production cannot be considered representative of the full annual cycle. They relate to a specific, transient stage of 4 months per year, while the overall community structure and metabolism of thermokarst lakes may be mostly defined by microbial characteristics during the prolonged winter season. These results also imply that accurate estimates of annual carbon fluxes and biogenic gas emissions from thermokarst lakes will require greater attention during the ice-cover and break-up periods.

### Winter Pathways of Permafrost Organic Carbon Degradation

Thermokarst lakes are enriched in terrestrial organic carbon, with up to 96% of the carbon of the DOM originating from terrestrial (thawed permafrost) origin and negligible contribution from phytoplankton and macrophytes (Wauthy et al., 2018). The high DOM concentrations measured in the present study during winter imply an abundance of permafrost-derived organic substrates for microbial degradation processes under the ice. Our metagenomic results indicate that the winter community had the potential for mobilizing and converting this complex organic matter to more labile carbon sources. For example, genes coding for hydrolytic arylsulfatases (*aslA* in Figure 3), which hydrolyse sulfate ester and release sulfate and phenols (Kertesz, 2000), or hydrolytic dehalogenases (*dhaA, had*), which cleave halogen groups from carbon substrates (Koudelakova et al., 2013), were abundant in the winter metagenomes and occurred in numerous genomic bins (Figures 3, 4). Sulfate released by sulfatase activity might be used by some members of the Deltaproteobacteria, Chloroflexi and Bacteroidetes that have the metabolic potential for sulfate and sulfite reduction (*dsr* and anaerobic sulfite reductase gene *asr* in Figure 4), contributing to the high concentrations of H_2_S measured in the water. The production of H_2_S and fermentation products such as fatty acids and CO_2_ likely explain the observed acidification of the water during winter. This acidification has important consequences, since it would also enhance the mobilization of labile carbon, iron, arsenate and selenate from colloidal and complexed organic matter (Audry et al., 2011; Pokrovsky et al., 2011). Metagenomic analysis showed a strong potential for the respiration of arsenate and selenate (*arrA* and *xdhD* in Figures 3, 4), suggesting that acidic mobilization of such elements could provide an additional energy source for permafrost-degrading microorganisms.

The main winter lineages (Aminicentantes, Bacteroidetes, Chloroflexi, Deltaproteobacteria, Planctomycetes and Verru- comicrobia) had a strong catabolic potential centered on the degradation of aromatic carbon and other plant detritus, which constitute the main organic matter of permafrost soils (Jansson and Taş, 2014). The catabolic potential of the reconstructed bins related to these lineages (average of 90 CAZy genes per bin) far exceeded the catabolic potential detected in bins associated with the summer community members (average of 42 CAZy genes per bin) and is consistent with the known properties of cultured representatives (Yamada et al., 2005; Wagner and Horn, 2006; Meckenstock et al., 2016) and previous genomic investigations (Hug et al., 2013; Tran et al., 2018; Woodcroft et al., 2018). However, different substrate specificities (cellulose, xylan, pectin, starch, esters, aromatic compounds) were detected among these winter lineages (Figure 4), suggesting multiple ecological niches and nutritional diversification. Considerable potential for the fermentation of catabolic products associated with mechanisms for hydrogen, formate and acetate transfer and utilization was identified in the microbial community, suggesting that syntrophic degradation and co-metabolism represent major pathways for the decomposition of permafrost organic carbon. This is also supported by the high *in situ* concentrations of fermentation intermediates identified in Arctic and subarctic peat (Tveit et al., 2015). These fermentative lineages are likely coupled with the methanogens (Figure 2) that consume hydrogen, as identified in a genomic bin of the Methanomicrobiales or acetate for the members of the Methanosarcinales (Jetten et al., 1992). This high potential for methanogenesis coupled with syntrophic degradation of the permafrost carbon is consistent with artificial thawing experiments (Tveit et al., 2015) and explains the elevated methane concentrations measured throughout the SAS2A water column in winter (Matveev et al., 2019).

In combination, our results indicate that catabolic diversification, fermentation pathways, syntrophic methanoge- nesis, and mobilization of recalcitrant organic matter and electron acceptors underpin the decomposition of permafrost-derived organic matter in lake SAS2A, and probably in many other thermokarst lakes. The detection of these processes implies a need for additional work. For example, although candidate phyla (*Parcubacteria*, *Microgenomates*) represented up to 15% of the total microbial community (Figure 2) and were detected throughout the year, suggesting a major role in the ecosystem, the metabolic functions of these taxa remain unclear. Genomic bin reconstruction highlighted that most of the enzymatic potential of these lineages differed from other bacterial lineages since only few genes were identified, as previously reported (Brown et al., 2015). The number of genes coding for carbohydrate degrading enzymes (CAZy in Figure 4) was low in Patescibacteria/CPR lineages, however, a potential for chitin or starch degradation was detected in the most complete genomic bins (Figure 4), indicating a possible role in permafrost carbon degradation.

## Conclusion

Previous work on thermokarst lakes has identified their importance for microbial activity and greenhouse gas production in northern landscapes, however, most studies to date have focused on the summer open water period. The present observations show that the winter microbiome differs greatly from that in summer in terms of both microbial composition and metabolic functions. The major shifts induced by prolonged ice cover in the winter (8 months per year) result in the seasonal partitioning of biogeochemical cycles, and favor the winter accumulation of methane and other reduced compounds. These results underscore the need for sampling thermokarst lakes in all seasons. They draw attention to the importance of winter at northern high latitudes, and show how this can be a period of diverse microbial pathways for the mobilization of organic matter and energy supply, notably fermentation processes, cross-feeding and syntrophic methanogenesis. Our observations imply that throughout much of the year, thermokarst lakes operate as cold, ice-capped methanogenic brews fuelled by permafrost-derived organic carbon.

## Data Availability

The datasets generated for this study can be found in NCBI, PRJNA515027.

## Author Contributions

AV, CL, AC, and WV designed the research. AV and PC analyzed the data with contributions from DK. AV, CL, PC, and WV led the writing of the manuscript. All authors provided comments on draft versions of the manuscript.

## Conflict of Interest Statement

The authors declare that the research was conducted in the absence of any commercial or financial relationships that could be construed as a potential conflict of interest.

## Footnotes

1 https://github.com/CruaudPe/MiSeq_Multigenique

2 http://bio-bwa.sourceforge.net
