# Supplementary material for: Contrasting Winter Versus Summer Microbial Communities and Metabolic Functions in a Permafrost Thaw Lake
Source: Front Microbiol. 2019 Jul 16;10:1656. doi: 10.3389/fmicb.2019.01656 (PMC6646835; doi:10.3389/fmicb.2019.01656)
Supplement: Supplementary file 1 [file Data_Sheet_1.docx]

Supplementary Material

## Supplementary Figures

**Supplementary Figure 1.** Microbial community composition determined by cDNA and DNA-based 16S rRNA gene amplicon sequencing. The right panel shows the bacterial community composition and the left panel the archaeal community composition in all summer (S1, S2, S3) and winter (W1, W2, W3) samples.

**Supplementary Material.** Metagenomes assembled genomes (bins) recovered and analyzed in this study.
